# Metabolic engineering design to enhance (R,R)-2,3-butanediol production from glycerol in *Bacillus subtilis* based on flux balance analysis

**DOI:** 10.1186/s12934-021-01688-y

**Published:** 2021-10-09

**Authors:** Nunthaphan Vikromvarasiri, Tomokazu Shirai, Akihiko Kondo

**Affiliations:** 1 RIKEN Center for Sustainable Resource Science, 1‑7‑22 Suehiro‑cho, Tsurumi‑ku, Yokohama, Kanagawa 230‑0045 Japan; 2 Department of Chemical Science and Engineering, Graduate School of Engineering, Kobe University, 1-1 Rokkodai, Nada, Kobe, 657-8501 Japan

**Keywords:** Glycerol, 2,3-Butainediol, *Bacillus subtilis*, Flux balance analysis, Genome-scale metabolic models, Gene knockout

## Abstract

**Background:**

Glycerol is a desirable alternative substrate for 2,3-butanediol (2,3-BD) production for sustainable development in biotechnological industries and non-food competitive feedstock. *B. subtilis*, a “generally recognized as safe” organism that is highly tolerant to fermentation products, is an ideal platform microorganism to engineer the pathways for the production of valuable bio-based chemicals, but it has never been engineered to improve 2,3-BD production from glycerol. In this study, we aimed to enhance 2,3-BD production from glycerol in *B. subtilis* through in silico analysis. Genome-scale metabolic model (GSM) simulations was used to design and develop the metabolic pathways of *B. subtilis*. Flux balance analysis (FBA) simulation was used to evaluate the effects of step-by-step gene knockouts to improve 2,3-BD production from glycerol in *B. subtilis*.

**Results:**

*B. subtilis* was bioengineered to enhance 2,3-BD production from glycerol using FBA in a published GSM model of *B. subtilis*, iYO844. Four genes, *ackA, pta, lctE,* and *mmgA*, were knocked out step by step, and the effects thereof on 2,3-BD production were evaluated. While knockout of *ackA* and *pta* had no effect on 2,3-BD production, *lctE* knockout led to a substantial increase in 2,3-BD production. Moreover, 2,3-BD production was improved by *mmgA* knockout, which had never been investigated. In addition, comparisons between in silico simulations and fermentation profiles of all *B. subtilis* strains are presented in this study.

**Conclusions:**

The strategy developed in this study, using in silico FBA combined with experimental validation, can be used to optimize metabolic pathways for enhanced 2,3-BD production from glycerol. It is expected to provide a novel platform for the bioengineering of strains to enhance the bioconversion of glycerol into other highly valuable chemical products.

**Supplementary Information:**

The online version contains supplementary material available at 10.1186/s12934-021-01688-y.

## Background

Biodiesel is a liquid biofuel derived from natural fats through a chemical reaction process (esterification or transesterification). Biodiesel has advantages over fossil diesel in terms of health and environmental concerns, such as lower sulfur and carbon dioxide emissions, which may contribute to a reduction in global warming. In 2019, 24% of the global carbon dioxide emissions from fossil fuel consumption were related to the transport section, and approximately 75% of the total carbon dioxide emissions from this section was from commercial vehicles [1]. Biodiesel not only provides the same high engine performance and calories as diesel, but it also has a higher flash point and better viscosity. These advantages have led to an increase in the use of biodiesel as a renewable fuel, offering an innovative solution to reduce the global air pollution caused by the increasing number of vehicles in Asian countries [2].

Biodiesel production generates large amounts of crude glycerol as a by-product; every ton of biodiesel produced generates approximately 100 kg of crude glycerol. Despite the increasing purification capacity and the development of new applications, biodiesel production has been associated with increased glycerol generation over the past few decades. Hejna et al. [3] reported that approximately 1 million tons of crude glycerol are produced annually from biodiesel production in Europe. Between 2015 and 2017, crude glycerol production increased from 1.16 to 2 million tons in Europe, and its production is expected to increase to more than 5 million tons by 2026 worldwide [4]. The large surplus of waste glycerol has affected the refined glycerol market and caused a significant reduction in the glycerol price. Researchers have attempted to convert glycerol into more useful products, such as 1,3-propanediol, succinic acid, and ethanol, in order to reduce the cost of biodiesel production and the toxic effects of waste glycerol on the environment [5].

Currently, sugar, starch, and molasses are the major raw materials for commercial biological products, such as proteins and biochemical monomers. Recent studies have focused on the use of renewable and non-food competitive materials as alternative resources; glycerol is a promising substrate for use as a renewable and low-cost substrate for biological production platforms.

(R,R)-2,3-Butanediol (2,3-BD) is a valuable bio-based chemical compound that has a wide range of potential applications, such as in the manufacturing of printing inks, antifreeze agents, foodstuffs, chemicals, and pharmaceuticals [6]. In addition, 2,3-BD can be used as a chemical substance for the production of more applicable chemicals, such as 1,3-butadiene, which can serve as a raw material for synthetic rubbers (methyl-ethyl-ketone), acetoin, and diacetyl, which can be used as liquid fuel additives, etc. [7]. 2,3-BD has roles in high-value manufacturing in various industries, such as the cosmetics, agricultural, and pharmaceuticals industries, and the bioprocess of 2,3-BD production has advantages over chemical processes in terms of environmental friendliness and lower cost [8]. Therefore, in this study, we focused on the conversion of glycerol into 2,3-BD using biological processes.

Among industrial microbes, *Bacillus subtilis*, a “generally recognized as safe” organism, is an ideal platform organism for engineering bacterial pathways for the biological production of various useful metabolites, such as lactate, acetate, acetoin, and 2,3-BD [9, 10]. *B. subtilis* is a superior host for bioproduction as compared to *Escherichia coli* because it is more tolerant to fermentation products, such as butanol and ethyl acetate [11], and lacks lipopolysaccharides, generally referred to as endotoxins, which are present in the outer cell membrane of *E. coli* and other gram-negative bacteria [12].

Bioinformatics tools have become increasingly attractive. Various techniques have been used to simulate and analyze biochemical systems. In silico genome-scale metabolic models (GSMs), which can be used to evaluate all metabolic reactions in a cell, are formulated on the basis of genome annotation data and experimental data. GSMs computationally describe gene-protein-reaction associations for a whole set of metabolic genes in an organism and can be used to simulate metabolic fluxes [13]. GSMs can predict metabolic flux using mathematical optimization techniques, such as flux balance analysis (FBA), through linear programming. Comparison of the predicted metabolic flux distributions under different conditions allows the evaluation of metabolic responses to identify the optimal conditions for a certain objective, such as growth rate [14]. FBA can also be applied to steady-state analysis of a given system using a stoichiometric matrix. When the objective function is resolved, the steady-state flux distribution can be obtained by solving a set of equations and is used to interpret the capacities of the metabolic system [15]. Several databases, including BsubCyc, KEGG, and NCBI, provide detailed information on the genome, genes, proteins, and metabolic and regulatory pathways of *B. subtilis*. Thus, in silico simulation with FBA is available for efficient metabolic pathway design of *B. subtilis* for glycerol assimilation and 2,3-BD production in a complex metabolic system. In preliminary experiments, we found that the *B. subtilis* model described by Oh et al. [16], iYO844 GSM, showed the most reliable performance for the simulation of growth on glycerol.

Previous bioengineering research on *B. subtilis* has focused mostly on improving the production of 2,3-BD from sugars such as glucose [17–19], not from glycerol. Only a few bioengineering studies have used glycerol for 2,3-BD production, e.g., in *Klebsiella oxytoca* [20], *Klebsiella pneumoniae* [21], and *Bacillus amyloliquefaciens* [22]. Bioengineering *B. subtilis* to improve 2,3-BD production from glycerol represents an alternative for applications in biorefineries. The iBsu1147 metabolic network model of *B. subtilis* has been used to generate an in silico metabolic engineering design of reactions to increase the production of 2,3-BD from glucose [23]; however, the design was not experimentally validated. To the best of our knowledge, this is the first study to use in silico FBA simulation to improve 2,3-BD production from glycerol in *B. subtilis* and evaluate the effects of step-by-step gene knockouts by validating the in silico design with experimental data.

The aim of this study was to enhance 2,3-BD production from glycerol in *B. subtilis* using in silico analysis to identify gene knockout targets in order to improve the metabolic pathway. A prototype of the metabolic pathways of *B. subtilis* for 2,3-BD production from glycerol was designed using the iYO844 model, considering growth in M9 medium. Changes in the bio-fermentation profiles caused by four gene knockouts are presented, and fermentation products were periodically collected and analyzed. Furthermore, the in silico analysis results were compared with experimental fermentation profiles.

## Results and discussion

### In silico simulation of 2,3-BD production from glycerol by *B. subtilis* strains

**Fig. 1:**
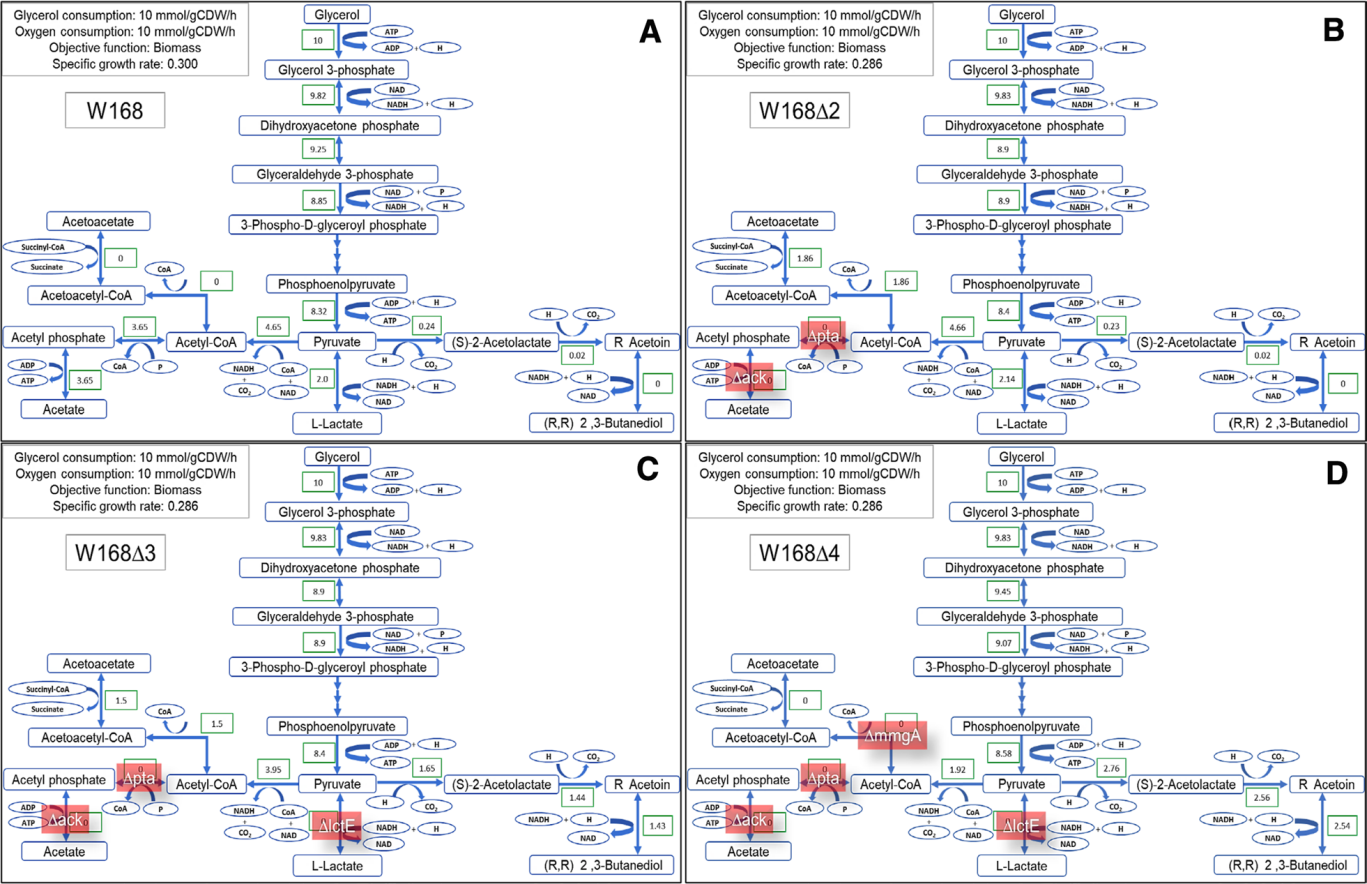
Metabolic pathways designs for 2,3-BD production from glycerol in *B. subtilis* (iYO844 model) based on FBA.** A**–**D**. Metabolic fluxes in *B. subtilis* wild-type strain 168 (W168), W168∆2, W168∆3, and W168∆4, respectively. Reaction substrates are indicated in blue boxes; conversion rates (unit: mmol/gCDW/h) in green boxes; and gene knockout reactions in red boxes

We modified and optimized the *B. subtilis* model iYO844 [16], which in a preliminary study provided the most reliable results for growth on glycerol, for in silico prediction of 2,3-BD production using FBA*.* Briefly, the environmental conditions in the iYO844 model were modified to the use of M9 medium with glycerol and an oxygen consumption rate of 10 mmol/gCDW/h, and biomass flux was the objective function. The regulation of glycerol uptake and metabolism in the model was optimized using the glycerol kinase (*glpK*) pathway (see “Methods” section). The primary results of glycerol utilization and metabolic flux for 2,3-BD production are shown in Fig. 1A. The biomass flux was 0.3 h^–1^, and acetic acid and lactic acid were the major fermentation products (3.65 and 2.0 mmol/gCDW/h). Fluxes of other volatile fatty acids (VFAs) were not observed.

To test the inhibitory effect of acetic acid production, the metabolic fluxes of the ACKr and PTAr reactions, which refer to the acetate kinase (encoded by *ackA*) and phosphate acetyltransferase (encoded by *pta*) reactions, were set to zero (Fig. 1B; Table 5). Thus, the flux of the PTAr reaction became zero, which led to zero flux in the ACKr reaction. However, a negligible amount of acetic acid (~ 0.12 mmol/gCDW/h) was produced from the essential metabolic reaction in iYO844 model, which is catalyzed by *N*-acetyldiaminopimelate deacetylase (BSU14190; abbreviation in the model: ADPDA) (see Additional file 1). The specific growth rate insignificantly decreased to 0.286 h^–1^. Lactic acid production slightly increased to 2.14 mmol/gCDW/h, acetoacetate production (ACACT1r and OCOAT1 reactions) increased from 0 to 1.86 mmol/gCDW/h, and the fluxes of other fermentation products were not affected. The results from this simulation showed that inactive ACKr and PTAr reactions did not promote or contribute to 2,3-BD production.

Next, we inactivated the LDH_L reaction to evaluate the effect of L-lactate dehydrogenase gene (*lctE*) deletion (Fig. 1C). The results showed that there was no change in the specific growth rate (0.286 h^–1^), whereas the LDH_L reaction flux became zero. The flux of acetoacetate production decreased to 1.5 mmol/gCDW/h. Interestingly, 2,3-BD production became a major by-product (1.43 mmol/gCDW/h), whereas the production of other VFAs remained zero. The results of this simulation clearly showed that suspension of the LDH_L reaction can enhance 2,3-BD production from glycerol in *B. subtilis*.

From the simulation results of the three inactivated reactions, we inferred that the major fermentation products were acetoacetate and 2,3-BD. Since the target of this study was 2,3-BD production alone, we had to eliminate the metabolic flux of acetoacetate production. The conversion of pyruvate into acetoacetate involves three reactions catalyzed by pyruvate dehydrogenase (PDH), acetyl-CoA C-acetyltransferase (ACACT1), and 3-oxoacid CoA-transferase (OCOAT1). The PDH reaction is an essential metabolic reaction that cannot be disabled, and the OCOAT1 reaction involves multiple genes, including BSU38990 and BSU38980. Therefore, the reaction catalyzed by ACACT1, encoded by *mmgA*, was a suitable target for inactivation (Fig. 1D). Growth was nearly unchanged (0.283 h^–1^), and the metabolic fluxes of the OCOAT1 and ACACT1 reactions became zero. As expected, the PDH flux was reduced by about half, and 2,3-BD production increased to 2.54 mmol/gCDW/h. Importantly, 2,3-BD was the only major product at this stage, whereas other products, such as ethanol, diacetyl, and propionic acid, were not detected. In the next phase of our study, the order of the simulations was applied to step-by-step gene modification in *B. subtilis* to validate the hypothesis from these simulations.

Hao et al. [23] used a *B. subtilis* GSM (iBsu1147) and flux variability analysis simulations to identify target reactions of genes to overexpress or underexpress to allow 2,3-BD production from glucose. The micro-aerobic condition was applied in the simulation (0.0005 mmol/gCDW/h), referring to the constrained oxygen condition at 0.5% saturated dissolution. Candidate reactions were predicted by using the flux variability analysis. The simulation results suggested that the first four genes to underexpress were *pyc* (encoding pyruvate carboxylase), *ytiB* or *yvdA* (encoding putative carbonic anhydrase), *ndk* (encoding nucleoside diphosphate kinase), and *gmk* (encoding guanylate kinase). These four genes are entirely different from the target genes and not related to those of the simulation for gene deletion in this study. Also, the biomass flux was zero when FBA simulation was conducted with knockout of these four genes in the iYO844 model. The difference in the prediction results may be due to the use of different models, environmental conditions, and prediction methods.

### Effects of gene knockouts on 2,3-BD production from glycerol in *B. subtilis* strains

**Fig. 2:**
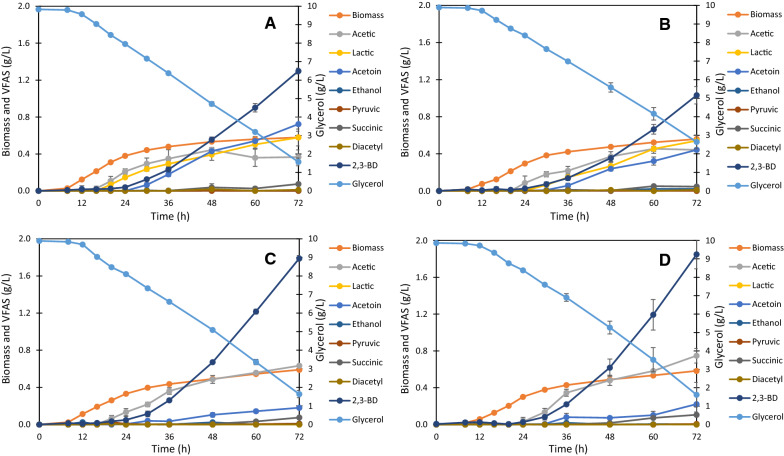
Time courses of growth, glycerol utilization, and VFAs production for *B. subtilis* strains as follows W168 (**A**), W168∆2 (**B**), W168∆3 (**C**), and W168∆4 (**D**). The data are presented as the mean ± standard deviation of three independent experiments (n = 3)

Fermentation experiments with four *B. subtilis* step-by-step knockout strains (W168, W168∆2, W168∆3, and W168∆4) were performed to validate the predictions based on the GSM simulation. The growth, glycerol utilization, and fermentation characteristics of each strain are shown in Fig. 2. The specific growth rate of W168 was 0.2 h^–1^, and the glycerol consumption rate was 7.26 mmol/gCDW/h. The fermentation profile of W168 (Fig. 2A) showed that the major fermentation products were acetic acid, lactic acid, acetoin, and 2,3-BD. Acetic and lactic acid production corresponded with growth, whereas acetoin and 2,3-butanediol production occurred during the stationary phase. Diacetyl, ethanol, pyruvic, and succinic acid production was not detected throughout the experiment. This result was similar to previous results of fermentation with *B. subtilis* W168 in M9 medium containing 10 g/L glucose under micro-aerobic conditions, in which 2,3-BD, acetoin, acetic acid, and lactic acid were the major fermentation products, succinate and ethanol accumulation were negligible, and pyruvic acid, α-ketoglutaric acid, and diacetyl were not detected throughout the fermentation process [24].

Figure 2B shows the effects of *ackA* and *pta* double knockout on growth, glycerol utilization, and VFAs production. When compared to W168, acetic acid production was delayed by 4 h in W168∆2; W168 produced acetic acid after 16 h of incubation, whereas W168∆2 produced acetic acid after 20 h of incubation. However, the major fermentation products were similar to those of W168, and acetic acid was still produced in significant amounts. In line herewith, Fu et al. [19] reported that *pta* inactivation hardly reduced acetate production in *B. subtilis*, which may be because of other pathways except acetate or acetyl-phosphate synthesis in *B. subtilis*. Besides acetic acid production by N-acetyldiaminopimelate deacetylase (*dapL*; BSU14190; EC:3.5.1.47) as mentioned in the previous section, we found that acetic acid can also be produced by N-acetylglucosamine-6-phosphate deacetylase (*nagA*; BSU35010; EC 3.5.1.25) and cysteine synthase (cysK, BSU00730, EC:2.5.1.47), which correspond to metabolic reaction of *B. subtilis* 168 in the Kyoto Encyclopedia of Genes and Genomes (KEGG; http://www.genome.jp/kegg/). Moreover, there is an indirect pathway of *B. subtilis* 168 in which pyruvate is converted into acetic acid via pyruvate oxidase (*ydaP*, BSU04340, EC:1.2.3.3) and acylphosphatase (*yflL*, BSU07640, EC:3.6.1.7).

The specific growth rate of W168∆2 was not different from that of W168 (0.2 h^–1^), but the glycerol consumption rate was higher (8.7 mmol/gCDW/h). In addition, some VFAs were produced at slightly lower final concentrations than by W168, which may be explained by the decrease in glycerol consumption after the exponential phase. These results indicated that knockout of *ackA* and *pta* did not improve 2,3-BD production (Table 1). The 2,3-BD yield of W168∆2 after 72 h of incubation was not increased compared to that of the wild-type strain.

**Table 1:** Yields of VFAs products from the *B. subtilis* strains after 72 h of incubation

Strain	VFAs^*^ (unit: mol/mol‧glycerol)				Glycerol Utilization (mol)
	Acetic acid	Lactic acid	Acetoin	2,3-BD	
W168	0.07 ± 0.014	0.07 ± 0.011	0.09 ± 0.009	0.16 ± 0.004	0.09 ± 0.002
W168∆2	0.09 ± 0.005	0.08 ± 0.011	0.06 ± 0.002	0.15 ± 0.013	0.08 ± 0.004
W168∆3	0.12 ± 0.001	0.00 ± 0.000	0.02 ± 0.002	0.22 ± 0.004	0.09 ± 0.002
W168∆4	0.14 ± 0.015	0.00 ± 0.000	0.03 ± 0.011	0.23 ± 0.019	0.09 ± 0.007

^*^The concentrations of diacetyl, ethanol, pyruvic, and succinic acid were < 0.01 mol/mol‧glycerol

The effects of *lctE* and *mmgA* deletion are shown in Fig. 2C and D. The specific growth and glycerol consumption rates of the W168∆3 and W168∆4 strains were similar to those of the W168∆2 strain. Thus, knockout of *lctE* and *mmgA* has no effect on the growth and glycerol uptake rates in *B. subtilis*. VFAs indicated that no significant amounts of diacetyl, ethanol, pyruvic, and succinic acid were produced, similar to the results for the wild-type and W168∆2 strains. *lctE* knockout caused inhibition of total lactic acid production and significantly suppressed acetoin production. Acetic acid was gradually produced during the stationary phase. Interestingly, *lctE* deletion had a noticeable favorable effect on 2,3-BD production; the 2,3-BD yield of W168∆3 reached 0.22 mol/mol‧glycerol, whereas that of W168 and W168∆2 was 0.16 and 0.15 mol/mol‧glycerol, respectively. These results were in line with the results of the in silico simulations, except for acetic acid production flux, which is discussed in the next section.

When considering the metabolic pathway of glycerol dissimulation to 2,3-BD, we found that the L-lactate dehydrogenase (BSU03050, *lctE*) reaction might compete with the (R,R)-butanediol dehydrogenase (BSU06240; *bdhA*) reaction for NADH, which is mainly produced by glycerol-3-phosphate dehydrogenase (BSU22830; *glpD*) and glyceraldehyde-3-phosphate dehydrogenase (BSU33940; *gapA*). Hence, the deletion of *lctE* eliminates the NADH competitive reaction, which results in an increase in 2,3-BD production from glycerol. In addition, *lctE* deletion has been reported to increase the production of 2,3-BD in other platforms, including *B. subtilis* [19], *K. oxytoca* [25], *Enterobacter cloacae* [26], and *K. pneumoniae* [27]. Therefore, *lctE* deletion can be regarded as key in improving 2,3-BD production in many microorganisms.

Remarkably, *mmgA* knockout caused an increase in 2,3-BD production. There is no previous study on the deletion of *mmgA* to enhance 2,3-BD production. The 2,3-BD yield of W168∆4 was 0.23 mol/mol‧glycerol. The product of *mmgA*, acetyl-CoA C-acetyltransferase (BSU24170, EC:2.3.1.9), is involved in the conversion of acetyl-CoA into acetoacetyl-CoA, which is a precursor of acetoacetate production for pyruvate and butanoate metabolism. Thus, *mmgA* deletion leads to the suppression of the metabolic flux from pyruvate to acetyl-CoA, which may contribute to an increase in the fluxes associated with 2,3-BD production. We conducted a micro-aerobic fermentation experiment to ensure the promotive effect of *mmgA* knockout on 2,3-BD production from glycerol. Under the same conditions, W168∆3 produced 1.93 ± 0.10 g/L 2,3-BD, whereas W168∆4 produced 2.13 ± 0.05 g/L after 96 h of incubation. This result confirmed that *mmgA* knockout contributed to the increase in 2,3-BD production.

The theoretical yield of the conversion of glycerol into 2,3-BD is 0.5 mol/mol glycerol [28]. The 2,3-BD yield of W168 strains were shown in Table 1. The maximum yield of 2,3-BD production by W168Δ4 (0.23 mol/mol glycerol) was 46% from the theoretical yield. The 2,3-BD yield of W168Δ4 was reduced by acetic acid production. M9 medium, which is a minimal medium, was used in this study to only provide the environment that we can control and connect to the GSM model. Therefore, the optimization of fermentation conditions (such as pH, glycerol concentration, temperature, etc.) to increase 2,3-BD production is necessary for further study. Furthermore, NADH was reported as an important key cofactor to improve the production of 2,3-BD from glucose and glycerol in *B. subtilis* and other microorganisms [22, 24, 28, 29]. The addition for the high availability of NADH contributes to enhancing 2,3-BD production by promoting the activity of 2,3-butanediol dehydrogenase rather than acetoin reductase.

### Comparison of the FBA results with experimental data

The specific growth rate (µ, h^–1^) and glycerol consumption rate (ν, mmol/gCDW/h) derived from the experimental data of the *B. subtilis* strains were applied in the in silico FBA simulation to determine the oxygen consumption rate (Table 2, Additional file 1). We found that the oxygen consumption rate did not significantly differ among the strains, demonstrating the similarity of the environmental conditions in the experiment.

**Table 2:** FBA of experimental data on the specific growth rate and glycerol consumption rate of the *B. subtilis* strains

Parameter (unit: mmol/gCWD/h)		*B. subtilis* strains			
		W168	W168∆2	W168∆3	W168∆4
Glycerol consumption (ν)^*^		– 7.26	– 8.70	– 8.70	– 8.70
*B. subtilis* biomass (µ, h^–1^)^*^		0.20	0.20	0.20	0.20
Oxygen consumption		– 7.27	– 7.35	– 7.35	– 7.40
Acetate exchange		2.83	0.09	0.09	0.09
L-Lactate exchange		1.62	3.70	0.00	0.00
(R,R)-2,3-butanediol exchange		0.00	0.00	2.47	2.77

^*^Experimental data

In the FBA, the highest acetic production rate was obtained for the W168 strain, which corresponded to the experimental result of acetic production of W168 during the exponential phase. However, in the FBA, after inactivation of the ACKr and PTAr reactions, the acetic production rate became 0.09 mmol/gCDW/h, which was not in line with the experimental findings after *ackA* and *pta* knockout. Although acetic production during the exponential phase of these strains was low, acetic acid production did occur during the stationary phase, likely because of the existence of alternative pathways of synthetic acetic acid in *B. subtilis*. The iYO844 model contains many reactions related to acetic production, but the FBA results of these reaction fluxes, including AGDC (N-acetylglucosamine-6-phosphate deacetylase), AHSERL3 (cysteine synthase), and ALDD2x (aldehyde dehydrogenase), were zero. Moreover, other reactions involved in acetic production, such as those catalyzed by pyruvate oxidase (BSU04340, EC:1.2.3.3) and acylphosphatase (BSU07640, EC:3.6.1.7), are not represented in this model. While we found that many reactions are missing the iYO844, this model provided the most reliable result of biomass production from glycerol, as mentioned above. Therefore, further development and improvement of the *B. subtilis* model are necessary to allow for more accurate in silico simulations in future. To date, substantial research has focused on reconstructing GSMs of *B. subtilis* as a representative model for other gram-positive bacteria [13].

The specific growth rates of the mutant strains were not different from that of W168 (0.2 h^–1^), whereas the glycerol consumption rates were higher (8.7 mmol/gCDW/h). However, the final concentrations of some of the major VFAs were slightly lower for W168∆2 than for W168, which may have been due to the decrease in glycerol consumption after the exponential phase. For *ackA* and *pta* knockout in W168∆2, the FBA results predicted that the fluxes of 2,3-BD production were zero. Indeed, knockout of these two genes did not affect 2,3-BD production (Table 2); the 2,3-BD production rates for W168 and W168∆2 were 0.61 and 0.60 mmol/gCDW/h, respectively.

FBA predicted that in W168∆3, the flux of lactic acid production would be zero as the LDH_L reaction was inactivated. Indeed, we found that lactic acid production was inhibited by *lctE* knockout. Moreover, the flux of 2,3-BD production increased to 2.47 mmol/gCDW/h in W168∆3, which was substantially higher than that of W168∆2 (Table 1). To assess the effect of *mmgA* knockout, FBA results of W168∆3 and W168∆4 on 2,3-BD production were compared. The FBA results predicted that the 2,3-BD production rate of W168∆4 would be slightly greater than that of W168∆3, which was in line the experimental result; the 2,3-BD production rate of W168∆4 was 1.06 mmol/gCDW/h, whereas that of W168∆3 was 0.96 mmol/gCDW/h.

We found that *acoA* (acetoin dehydrogenase, BSU08060) was knocked out in previous studies in order to enhance 2,3-BD production in *B. subtilis* [19, 24]. However, the FBA simulation in this study did not suggest an ethanol production flux from this reaction. Moreover, the fermentation results of *B. subtilis* W168 in M9 medium containing glucose or glycerol did not indicate ethanol to be a significant product. Therefore, knockout of *acoA* was not required. This demonstrates that the use of in silico simulation can save time, energy, labor, and costs when compared to trial-and-error experiments.

Although the FBA results were not exactly the same as the experimental results, the guidance of gene knockout based on in silico simulations was very accurate, and the trends in the simulation results were consistent with the experimental results. 2,3-BD production from glycerol by the modified *B. subtilis* strains was substantially higher than that of the wild-type strain. The promotive effect of *mmgA* knockout on 2,3-BD production from glycerol, which was predicted based on the FBA simulation results, was demonstrated for the first time in this study.

## Conclusions

Glycerol is a renewable and low-cost substrate that has potential as a raw material for industrial biological production processes. 2,3-BD is a valuable bio-based chemical compound for a wide range of potential applications and was selected as the target product in this study. The iYO844 genome-scale metabolic model of *B. subtilis* was applied to design and develop the metabolic pathways. FBA results provided precise target reactions to guide gene knockouts to improve the metabolic pathway for 2,3-BD production from glycerol. Furthermore, the use of in silico simulation of FBA could provide a novel platform solution for the bioengineering of *B. subtilis* to enhance 2,3-BD production from glycerol, which was the guidance of *mmgA* knockout. Additionally, the prediction results can save time and costs from unnecessary gene knockouts. The methodology of this study can be applied to increase other target fermentation products through the use of FBA in GSMs of other microorganisms. We are currently focusing on the development of GSMs and prediction strategies to provide better simulation results in future studies. The experimental knowledge and updated information derived from these experiments can be used to develop accurate and efficient models and predictions.

## Methods

### Strains and plasmid construction

The strains and plasmids used in this study are listed in Table 3. The parent strain, *B. subtilis* wild-type 168 (W168), was obtained from the Microbe Division at the RIKEN BioResource Research Center, Japan. We used the CRISPR-Cas9 system for gene knockout in *B. subtilis* [30]. Plasmid pJOE8999 was obtained from the Bacillus Genetic Stock Center (Ohio State University, Columbus, OH, USA). For plasmid construction, we used *Escherichia coli* NovaBlue (Novagen, Cambridge, MA, USA).

**Table 3:** Strains and plasmids used in this study

Strain or plasmid	Genotype or relevant characteristic	Reference
Strains		
* E. coli* Nova blue	*endA1 hsdR17*(r_*K12*_^−^m_*K12*_^+^) *supE44 thi-I gyrA96 relA1 lac recA1*/F’[proAB + lacIq ZΔM15::Tn10(Tetr)]	Novagen
* B. subtilis* W168	*trpC2*	JMC^a^
* B. subtilis* W168∆2	*trpC2∆ackA∆pta*	This study
* B. subtilis* W168∆3	*trpC2∆ackA∆pta∆lctE*	This study
* B. subtilis* W168∆4	*trpC2∆ackA∆pta∆lctE∆mmgA*	This study
Plasmids		
pJOE8999	Kan^R^, P_*manPA*_-*cas9*, P_vanP*_, *lacPOZ’*-gRNA, *oop ter*, T7P, *repE194*^*ts*^, pUC*ori*	Altenbuchner [30]
pJOE_ackA	pJOE8999 derivative, containing 20-nt spacer targeting *ackA* and fused homologous arms of *ackA*	This study
pJOE_pta	pJOE8999 derivative, containing 20-nt spacer targeting *pta* and fused homologous arms of *pta*	This study
pJOE_lctE	pJOE8999 derivative, containing 20-nt spacer targeting *lctE* and fused homologous arms of *lctE*	This study
pJOE_mmgA	pJOE8999 derivative, containing 20-nt spacer targeting *mmgA* and fused homologous arms of *mmgA*	This study

^a^Microbe Division/Japan Collection of Microorganisms (JMC), RIKEN BioResource Research Center

For plasmid construction for *ackA* gene knockout (pJOE_ackA), plasmid pJOE8999 was cut at the *Bsa*I restriction site and purified by gel purification (Wizard® SV Gel and PCR Clean-up System kit, Promega, WI, USA). The primers used in this study are listed in Table 4. An oligonucleotide encoding a 20-nucleotide sgRNA with flanking *Bsa*I sites (P_ackA01 and P_ackA02 primers) was introduced into pJOE8999 by polymerase chain reaction (PCR) using KOD FX Neo DNA polymerase (Toyobo, Osaka, Japan). The PCR product was digested with *Dpn*I restriction enzyme to remove the methylated DNA templates and gel-purified. Plasmid pJOE8999 containing a 20-nucleotide sgRNA for *ackA* deletion was cut using the *Sfi*I restriction enzyme and purified. Meanwhile, two homology arms for *ackA* deletion were PCR-amplified from *B. subtilis* W168 chromosomal DNA using the P_ackA03 and P_ackA04, and P_ackA05 and P_ackA06 primers. After PCR product purification, these two PCR fragments were combined by overlap extension PCR using the P_ackA03 and P_ackA06 primers. The overlap extension PCR fragment was integrated into the *Sfi*I site of the vector using NEBuilder HiFi DNA Assembly Master Mix (New England Biolabs, Ipswich, MA, USA), and transformed into *E. coli* NovaBlue for plasmid construction and replication. Transformants were selected on 25 μg/mL kanamycin in Luria–Bertani (LB) medium. pJOE_pta, pJOE_lctE, and pJOE_mmgA were constructed using the same protocol as that used for pJOE_ackA plasmid construction.

**Table 4:** Primers used in this study

Name	Sequence (5′ → 3′)	Usage
P_ackA01	CGGTATCGGTGAAAACAGTGGTTTTAGAGCTAGAAATAGCAAGTT	Construction of pJOE_ackA sgRNA
P_ackA02	GAGAATTGAGTAAAATGTACCTACGCGGTATCGGTGAAAACAGTG	
P_ackA03	GGTCGACGGCCAACGAGGCCCCCCTCTCAACCGTGTTTCTATTTT	PCR of homology arms for *ackA* deletion
P_ackA04	TCAAGAGAATGTGCTTTCATGCGATGATTGACGCTCCTTTATACTCTGTA	
P_ackA05	TCAAGAGAATGTGCTTTCATGCGATGATTGACGCTCCTTTATACTCTGTA	
P_ackA06	ATGAGCGAAGGAATGGCCTTCGTGGCCAATAAGGCCTTTCTAG	
P_ackA07	CGACGGAAGTATCAAGACCTCCTGA	PCR of check *ackA* deletion
P_ackA08	ACTGGCAGATCACAAATGACCGTG	
P_pta01	GAATTGAACCTTACGCTGGGCGGGTTTTAGAGCTAGAAATAGCAAGTT	Construction of pJOE_pta spacer
P_pta02	GAGAATTGAGTAAAATGTACCTACGGAATTGAACCTTACGCTGGGCGG	
P_pta03	GGTCGACGGCCAACGAGGCCTGGATACAGCTTTTGCAGCT	PCR of homology arms for *pta* deletion
P_pta04	GAGCTGCCATTGTCTTCAATTTTAAATAAAACCTCCTCA	
P_pta05	TTGTCTTCAATTTTAAATAAAACCTCCTCAAAAAGTTACAA	
P_pta06	GGCCAAAAAGCTGATTTTATGGGCCAATAAGGCCTTTCTAG	
P_pta07	GTCCCGGAGCCTTTCTCTCT	PCR of check *pta* deletion
P_pta08	GAAGATCTGGCAACGACAAC	
P_lctE01	GATGTGATGGATTTAAACCAGTTTTAGAGCTAGAAATAGCAAGTT	Construction of pJOE_lctE spacer
P_lctE02	GAGAATTGAGTAAAATGTACCTACGGATGTGATGGATTTAAACCA	
P_lctE03	GGTCGACGGCCAACGAGGCCCTTTCAATGATCAACTGACGATTAC	PCR of homology arms for *lactE* deletion
P_lctE04	GAAACATACCCTGGAAGGATGATCCGCAACTTTAGAGTAAAGGG	
P_lctE05	GAAACATACCCTGGAAGGATGATCCGCAACTTTAGAGTAAAGGG	
P_lctE06	GAATCAAACAGACATGGCCCGGCCAATAAGGCCTTTCTAG	
P_lctE07	TGTAGCTGTGCTTTATCCTGATGATATTGC	PCR of check *lactE* deletion
P_lctE08	TGCTCGGGCCGGAATTAGCAAACATTTT	
P_mmgA01	CTGAATGAGGTGAAACCATGGTTTTAGAGCTAGAAATAGCAAGTT	Construction of pJOE_mmgA spacer
P_mmgA02	GAGAATTGAGTAAAATGTACCTACGCTGAATGAGGTGAAACCATG	
P_mmgA03	GGTCGACGGCCAACGAGGCCTCGGATGATTCATGCCCAGC	PCR of homology arms for *mmgA* deletion
P_mmgA04	TTCAAATCCCCTTTTTCATCATGTTGGTTTCACCTCATTCAGAAGATAGA	
P_mmgA05	TTCAAATCCCCTTTTTCATCATGTTGGTTTCACCTCATTCAGAAGATAGA	
P_mmgA06	TCCTTTAAGTGATGTCGTGCGATTAGGCCAATAAGGCCTTTCTAG	
P_mmgA07	GGCCTGAGTGAAGGACTTCCATAAT	PCR of check *mmgA* deletion
P_mmgA08	ATCAAGCTCTATCCCATCCGCTCCG	

Plasmids pJOE_ackA, pJOE_pta, pJOE_lctE, and pJOE_mmgA were sequentially introduced into *B. subtilis* W168 by natural transformation [31] for multiple gene deletions in the order of the in silico simulation. Cells were plated on LB agar containing 5 μg/mL kanamycin and 0.2% mannose to introduce *cas9* under the control of P_mamP_. The plate was incubated at 30 °C for 2 days, and colonies were selected and transferred to LB agar without antibiotics and incubated at 50 °C overnight. The replication of pJOE8999 derivatives was temperature-sensitive, so the plasmid was removed during this process. The colonies were streaked on LB agar and incubated at 42 °C to obtain single colonies. The colonies were checked for target gene deletion by PCR using the above primers; for example, P_ackA07 and P_ackA08 primers were used to check for *ackA* deletion. Loss of the plasmid was evaluated by plating single colonies onto LB agar with and without kanamycin. Finally, target gene deletion was confirmed using DNA sequencing.

### Culture media and fermentation conditions

LB liquid medium contains (per L) 10.0 g tryptone, 5.0 g yeast extract, and 10.0 g sodium chloride, and for plates, 1.5% bacto-agar is added for solidification. LB medium was used for the maintenance and preservation of all strains, and for plasmid construction. All strains were preserved at − 80 °C and revived by inoculation on LB agar plates.

M9 medium [32] containing 10 g/L of glycerol was used as the minimal medium to investigate the bio-fermentation profile of all *B. subtilis* strains. Colonies of *B. subtilis* strains were cultured in 5 mL of M9 medium at 37 °C under rotation at 180 rpm overnight. Then, 1% (v/v) of the culture medium was activated by culture in 5 mL of M9 medium at 37 °C and 180 rpm for 18 h. Subsequently, 0.5% (v/v) liquid culture was inoculated into a 300-mL flask with 150 mL of M9 medium at 37 °C and 180 rpm for 72 h. Bacterial growth was periodically observed by measuring the optical density of the liquid culture at 600 nm (OD_600_). At the same time, liquid samples were collected and analyzed for residual glycerol and fermentation products, including acetic acid, lactic acid, pyruvic acid, succinic acid, diacetyl, acetoin, 2,3-BD, and ethanol. To confirm the effect of *mmgA* knockout on 2,3-BD production, micro-aerobic fermentation was performed using 120 mL of M9 medium at 37 °C and 100 rpm for 96 h.

### Analytical methods

The growth of *B. subtilis* strains was periodically observed by measuring the OD_600_ using a UVmini-1240 spectrophotometer (Shimadzu, Kyoto, Japan). The cell dry weight (CDW) was converted from the OD_600_ value; 1 OD_600_ is equal to 0.325 of g CDW/L [19]. The concentrations of glycerol and VFAs (acetic acid, lactic acid, pyruvic acid, succinic acid, diacetyl, acetoin, 2,3-BD, and ethanol) in the culture supernatant were monitored using a high-performance liquid chromatography instrument (Shimadzu, Japan) equipped with a UV/RI detector and an Aminex HPX-87H column (300 × 7.8 mm. (Bio-Rad, USA). A 10-μL sample was injected into the chromatography system operated at 55 °C and was eluted with 5 mM H_2_SO_4_ at a flow rate of 0.5 mL/min.

### In silico simulations

The GSM of *B. subtilis* strain 168 in this study referred to the iYO844 model constructed by Oh et al. [16]. This model was updated on October 31, 2019, and is available on the BiGG Model website (http://bigg.ucsd.edu/). The last version of the iYO844 model contained 990 metabolites, 1250 metabolic reactions, and 844 genes. For simulations to determine the metabolic flux distributions, FBA was performed with the commercial GLPK (GNU Linear Programming Kit) package, which supplies a solver for large-scale linear programming (LP) and mixed integer programming (MIP), and the COBRA toolkit [33] using MATLAB software (MathWorks, Inc., USA). M9 minimal medium for *B. subtilis* was applied by setting a lower bound to allow unlimited uptake on the exchange reactions of the necessary minerals. The ALCD19_D and ALCD19_L reactions, which involve alcohol dehydrogenases, were deleted from the model because of the determined roles of YhdN in methylglyoxal resistance [34].

To evaluate the growth and metabolic pathways of glycerol utilization as the sole substrate in the simulation of *B. subtilis* strains under limited oxygen conditions, the glycerol and oxygen consumption rates were set at 10 mmol/gCWD/h (the maximum uptake rate), and the biomass objective function was set to maximize the target product for the growth simulation. Gene knockout simulations were performed by setting the minimum and maximum fluxes of the related reactions of the target genes to zero. The available reactions for gene knockout did not include exchange reactions, transporter reactions, or essential gene reactions. Details of the gene targets for knockout are listed in Table 5. W168 corresponds with the *B. subtilis* wild-type strain 168, which was originally used in the iYO844 model. The modified strains correspond with the gene knockout strains of W168: W168∆2 (deletion of the ACKr and PTAr reactions), W168∆3 (deletion of the ACKr, PTAr, and LDH_L reactions), and W168∆4 (deletion of the ACKr, PTAr, LDH_L, and ACACT1r reactions).

**Table 5:** Information on target genes for knockout in the iYO844 model

Gene-protein-reaction	Gene^a^	Enzyme	Abbreviation in model^b^	EC number
BSU29470	*ackA*	Acetate kinase	ACKr	2.7.2.1
BSU37660	*pta*	Phosphate acetyltransferase	PTAr	2.3.1.8
BSU03050	*lctE*	L-lactate dehydrogenase	LDH_L	1.1.1.27
BSU24170	*mmgA*	Acetyl-CoA C-acetyltransferase	ACACT1r	2.3.1.9

^a^Data from the National Center for Biotechnology Information (NCBI), USA refers to the sequence: NC_000964.3

^b^iYO844 model referred to Oh et al. [16]

The specific cell growth rate (μ, h^−1^) and glycerol consumption rate (ν, mmol glycerol/gCDW/h) were evaluated from experimental data during the logarithmic phase using Eqs. 1 and 2, respectively. The necessary data for these calculations are cell density (X, g CDW/L), residual glycerol concentration (S, mmol/L), and time (t, h). The calculation results were applied to the FBA simulation for comparison.

1 $$\mu = \frac{\ln X1 - \ln X0}{{t1 - t0}}$$

2 $$\nu = \frac{{S_{1} - S_{2} }}{{\frac{1}{2}\left( {X_{2} + X_{1} } \right) \times \left( {t2 - t1} \right) }}$$

After deriving the experimental results of the specific cell growth and glycerol consumption rates, FBA simulations of each strain were performed again to compare the in silico results with the experimental results. The biomass flux was used as an objective function. Glycerol consumption rates were fixed according to the experimental results of each strain, and oxygen consumption rates were varied to obtain the specific cell growth rates based on the experimental results. The production of the target VFAs was compared between the experimental results and FBA simulation results.

## Supplementary Information

**Additional file 1.** FBA analysis results from the experimental data.

## Data Availability

All data generated or analysed during this study are included in this published article [and its supplementary information files].

## Abbreviations

2,3-BD: (R,R)-2,3-butanediol
CDW: Cell dry weight
FBA: Flux balance analysis
GSMs: Genome-scale metabolic models
LB: Luria–Bertani
PCR: Polymerase chain reaction
sgRNA: Single-guide RNA
VFAs: Volatile fatty acids
